# AMYPdb: A database dedicated to amyloid precursor proteins

**DOI:** 10.1186/1471-2105-9-273

**Published:** 2008-06-10

**Authors:** Sandrine Pawlicki, Antony Le Béchec, Christian Delamarche

**Affiliations:** 1 Université de Rennes I and CNRS UMR 6026, Equipe Structure et Dynamique des Macromolécules, Campus de Beaulieu, Nb 13, 35042 RENNES Cedex, France

## Abstract

**Background:**

Misfolding and aggregation of proteins into ordered fibrillar structures is associated with a number of severe pathologies, including Alzheimer's disease, prion diseases, and type II diabetes. The rapid accumulation of knowledge about the sequences and structures of these proteins allows using of *in silico *methods to investigate the molecular mechanisms of their abnormal conformational changes and assembly. However, such an approach requires the collection of accurate data, which are inconveniently dispersed among several generalist databases.

**Results:**

We therefore created a free online knowledge database (AMYPdb) dedicated to amyloid precursor proteins and we have performed large scale sequence analysis of the included data. Currently, AMYPdb integrates data on 31 families, including 1,705 proteins from nearly 600 organisms. It displays links to more than 2,300 bibliographic references and 1,200 3D-structures. A Wiki system is available to insert data into the database, providing a sharing and collaboration environment. We generated and analyzed 3,621 amino acid sequence patterns, reporting highly specific patterns for each amyloid family, along with patterns likely to be involved in protein misfolding and aggregation.

**Conclusion:**

AMYPdb is a comprehensive online database aiming at the centralization of bioinformatic data regarding all amyloid proteins and their precursors. Our sequence pattern discovery and analysis approach unveiled protein regions of significant interest. AMYPdb is freely accessible [1].

## Background

Amyloid deposits are abnormal *in vivo *extracellular aggregates of insoluble proteinaceous fibers exhibiting a cross-beta structure. The proteins or fragments found in these aggregates derive from diverse full-length precursors belonging to families without any obvious functional or structural resemblance. In addition to these quite typical extracellular deposits, other proteins can also form intracellular inclusions. Under the effect of diverse modifications, including interaction with chaperones, mutations, supraphysiological concentrations, post-translational modifications, and so on, amyloid proteins fail to fold properly, thus accumulating irreversibly over long periods, with toxic effect [2-4].

Protein misfolding is associated with a wide range of human diseases called amyloidoses. These may affect multiple tissues, in the case of systemic amyloidoses, or can be limited to a particular organ. Those pathologies may have major health and social impacts, as in the case of Alzheimer's disease [5], or might be somewhat benign, such as the amyloidosis that can occur among diabetics at the site of their insulin injections [6].

Prions are a special case among amyloid proteins because of their unusual properties. They originate from the conversion of a normal host protein into a fibrillar structure that then acts as an infectious particle [7]. To date only one prion, PrP, has been discovered in vertebrates. It is involved in major neurodegenerative diseases including Creutzfeldt-Jakob disease, Gerstmann-Straüssler-Scheinker syndrome, and Kuru in humans, scrapie in sheep, and spongiform encephalopathy in cattle. Prion proteins are also described in eukaryotic microorganisms (yeasts and fungi). However, in these latter organisms, the prion isoform is not always toxic and can control normal cellular processes [8,9]. The prion concept has been recently extended to include mammalian prion-like proteins, such as Tia-1. This is an RNA-binding protein implicated in the assembly of the cytoplasmic aggregates known as stress granules [10].

Schematically, the conversion of a normal soluble protein into insoluble amyloid fibers begins with a conformational change, resulting in an intermediate form, an amyloidogenic isoform. This new conformation favors self-association in small oligomers that act as nucleation units. The growth of the nucleation units leads to the formation of long protofilaments, which are wrapped to form mature fibers [11]. Biophysical techniques have shown that protofilaments may have various morphologies, but that they share common properties at the molecular level. The amyloid proteins/peptides form either parallel or anti-parallel arrangements of beta-strands. Since these beta-strands are perpendicular to the fiber axis, this has been described as a cross-beta structure [12]. Despite the difficulties of using experimental approaches to determine the precise 3D-structure of amyloid proteins in their fibrillar state, several models have recently been proposed [13]. These discoveries profit from computer simulations being used more and more often in biology.

Some authors have demonstrated that amyloid-like structures can be obtained *in vitro *with almost any protein, suggesting that the ability to form fibers is a common property of polypeptide chains [14]. However, the number of proteins aggregating *in vivo *is low compared to the over 3 million sequences stored in the Universal Protein Ressource (UniProtKB), and only include a few specific members of 31 families. The propensity of a protein to aggregate into amyloid fibrils varies greatly with the amino-acid sequence and with cellular environment. To take just two examples: the globular protein lysozyme is only associated with amyloid deposition in the kidney when it presents site-specific mutations (I56T, F57I, W64R, D67H) [15], while phosphorylation of Huntingtin may modulate its cleavage and toxicity [16].

During the past few years, bioinformatic approaches have been dedicated to the discovery of sequence segments that are sensitive to self-aggregation or that promote protein destabilization [14,17-27]. All methods presented in these papers are based on similar ideas. Each one tries to calculate various aggregation indexes and profiles by exploiting the information found in kinetic data, peptide/protein sequences, conformation space, and/or 3D-structures. However, a common problem encountered in these *in silico *experiments is the difficulty of finding and extracting accurate data from the existing literature and various molecular databases.

For studies focusing on sequence features, three databases are usually particularly useful: the UniProtKB, which provides sequences with functional annotations, comments, and cross-references [28]; the PROSITE database, which consists of a large collection of sequence signatures [29]; and the bibliographical database MEDLINE [30]. However, extraction of information from such general databases can be complex and time-consuming due to the large amount of data available and because of the diversity of the gene or protein families. To compensate for this, Siepen [31] developed a specialized database, fibril_one, dedicated to the analysis of mutations associated with fibrillogenesis. Unfortunately, the usefulness of this resource is limited, as fibril_one contains few data and has never been updated.

To facilitate *in silico *comparison of proteins involved in the formation of beta-sheet-rich fibrils *in vivo*, we have created a new multi-user database, the AMYloid Protein database (AMYPdb). The main goal in developing this relational database was to provide a regularly updated access to protein sequences and patterns describing each family. The 3,621 amino acid sequence patterns stored in the database can be screened to facilitate the assigning of new sequences to a particular family and the formulation of hypotheses about their function(s). Patterns conserved in several families may also help in extracting rules about the mechanisms of fibril formation.

## Results and Discussion

### Working with AMYPdb

AMYPdb is freely accessible [1]. Users can browse web pages to obtain descriptions of each family, visualize protein sequences enriched with links to both UniProtKB and the Protein DataBank (PDB), study multiple sequence alignments using the Jalview editor [32], or access bibliographic references. An identity card for each protein is available from the "protein menu". Links to Wikipedia provide further information on some families. Sequences can be selected and exported in FASTA format for further analysis.

The amino acid sequence patterns are accessible by browsing the pages from the "pattern" menu, or by using the search interface. The search page contains several menus, allowing the user to focus on particular data. For instance, they can interrogate AMYPdb with UniProtKB or PROSITE identifiers or patterns to determine whether a particular protein or pattern is stored in AMYPdb. They can also submit a personal signature to find any matching amyloid proteins, or inversely, they can submit a sequence to find matching AMYPdb patterns. It is also possible to select patterns using thresholds on quality scores. This method is useful for discovering patterns shared between families.

Beyond functioning as a pure repository of knowledge, AMYPdb also provides private workspaces to anyone interested in further analysis. This allows users to manage their own working sets of proteins and patterns, which can be easily manipulated and organized accordingly to their research interests.

Below, we have illustrated several ways that AMYPdb can be useful in pattern research on amyloid proteins.

### Protein family signatures

AMYPdb patterns can be used to highlight residues though to be important to the structure, function and evolution of protein families. One of the objectives of our project was to propose a list of specific amino acid sequence patterns for each family stored in the database. On the web interface of AMYPdb, the 3,621 amino acid sequence patterns are classified by their CF value. About 27% of the patterns have a CF ≥ 0.9 (956 of 3,621) and can be considered as very good descriptors for the corresponding amyloid families (Table 1, column n°5). The best results are shown in Table 2. It is interesting to note that all of the AMYPdb patterns noted in that table are of better quality than those in the PROSITE database.

**Table 1:** Amyloid families in AMYPdb

Family	Function	Pathology	S	A	P
Alpha Fibrinogen	Involved in the coagulation cascade	Autosomal dominant hereditary hepatic or renal amyloidosis	59	3	1
Alpha Synuclein	Unknown	Synucleopathies, such as Alzheimer's and Parkinson's diseases	21	85	0
Amyloid Beta Precursor	Protease inhibitor	Alzheimer's disease and aged Down's syndrome	125	108	3
Apolipoprotein A-1	Lipid metabolism	Autosomal dominant systemic amyloidosis	54	16	0
Atrial Natriuretic Factor	Blood pressure and sodium balance	Isolated Atrial Amyloid	34	5	1
Beta-2 Microglobulin	Class 1 human leukocyte antigen	Aggregation in the musculoskeletal system	141	0	1
Bri2	Unknown	Familial British Dementia (FBD)	3	*	0
C-Protein	Major component of lung surfactant	Pulmonary alveolar proteinosis	16	7	1+
Calcitonin	Polypeptidic hormone	Amyloid deposits in case of medullary thyroid cancer	37	16	1
Cystatin C	Cystein protease inhibitor	Alzheimer's disease and cerebral amyloid angiopathy	67	0	1
Gelsolin	Modulation of actin filament length	Gelsolin familial amyloidosis	39	18	0
Huntingtin	Fast axonal trafficking	Huntington's disease	19	109	0
Immunoglobulins	Immune response	Light-chain amyloidosis	0	*	0
Insulin	Metabolism of carbohydrates and fat	Localized amyloidosis at injection sites of type 1 diabetic patients	160	3	1
Islet Amyloid Polypeptide	Glycaemia regulation	Aggregates in pancreatic islets of type 2 diabetes and insulinomas	17	82	1
Lactadherin	Anticoagulant?	Aortic medial amyloidoses	5	*	3
Lactoferrin	Transferrin	Amyloid deposits in the cornea, seminal vesicles and brain	34	94	3
Lysozyme	Bacteriolytic enzyme	Non-neuropathic systemic amyloidosis	122	30	1
Microcin E492	Bacterial bacteriocine	Regulation of the protein's activity	2	*	0
Parkin	Proteasomal degradation?	Parkinson's disease	16	*	0
Prolactin	Hormone secreted by the pituitary gland	Amyloid deposits in pituitary glands of aging individuals	112	54	2
Serpin	Serine protease inhibitors	Serpinopathy	3	*	0
Serum amyloid A	Cell adhesion, migration, and proliferation	Inflammation-associated reactive amyloidosis	74	63	1+
Tau	Microtubule assembly and stability	Alzheimer's disease and dementias	37	13	1
Transthyretin	Thyroxine transport	Familial amyloid polyneuropathies	54	97	1+
Het-S	Heterocaryon incompatibility	Prionization involved in the protein's normal function	1	*	0
New 1	Unknown	No stable prion has been shown *in vivo*	2	*	0
Prion Protein (PrP)	Signal transmission, copper regulation?	Transmissible spongiform encephalopathies and dementias	353	6	2+
Rnq 1	Unknown	Unknown	1	*	0
Sup35	Translation termination factor	Prionization might be advantageous in stress conditions	82	48	1
Ure2	Nitrogen metabolism	Loss of Ure2 function	15	99	0

The six last lines of the table show the prion families. S, sequences in AMYPdb (full length and fragments); A, AMYPdb patterns with CF ≥ 0.9; *, not submitted to pattern discovery method; P, PROSITE super family signatures; P+, PROSITE family signatures.

**Table 2:** Best patterns describing amyloid protein families

Family		*CF*	Best pattern
Alpha Fibrinogen	A	0.91	C-x(7,8)-C-x(3)-[DGHMNPS]-W-[DGHMNPS]-x-K-C-P
	P	0.13	W-W-[LIVMFYW]-x(2)-C-x(2)-[GSA]-x(2)-N-G
Alpha Synuclein	A	1	S-[KR]-T-K-E-G-V-V-H-G
Amyloid Beta Precursor	A	1	S-x(0,3)-N-[KV]-[GP]-A-[IV]-[AI]-[DEG]-[EL]-[IM]-[QV]-[DG]-[EG]-V-[DV]-[EI]-[AL]
	P	0.75	G-Y-E-N-P-T-Y-[KR]
Apolipoprotein A-1	A	0.97	V-[HKR]-x-K-x-[DEGHKNPQRSTY]-[ENPQTV]-x-L-[DE]-[DEHNPQSWY]-[FILMVY]-[DEGHKNPQRSTY]-x-[EHIKLMQV]-x(2)-[ENPQTV]-[DEHKNPQY]-[ACHILMV]
Atrial Natriuretic Factor	A*	0.98	C-F-G-x-[KR]-[ILM]-D-R-I-G-[ANST]-x-S-[GS]-[LM]-G-C-[GNS]-[GNPRS]
	P	0.53	C-F-G-x(3)-[DEA]-[RH]-I-x(3)-S-x(2)-G-C
Beta-2 Microglobulin	A	0.71	P-x(2,3)-Q-[ETV]-[DGY]-[PST]-[ER]-x-[PW]-x-[DENQS]-x-[DGNT]-[DEKRT]-x-[NT]-x-[AILV]
	P	0.23	[FY]-{L}-C-x-[VA]-{LC}-H
C-Protein	A	1	R-L-L-[IV]-[AIV]-[VY]-[KV]-[PV]-[AIV]-[PV]
	P^+^	0.95	I-P-C-C-P-V
Calcitonin	A*	1	L-S-T-C-[MV]-L-[GS]-x-[LY]-[STW]
	P	0.53	C-[SAGDN]-[STN]-x(0,1)-[SA]-T-C-[VMA]-x(3)-[LYF]-x(3)-[LYF]
Cystatin C	A	0.68	V-x(2,6)-Q-x(1,2)-V-[AS]-G-x(2)-[HY]-[FIKRY]-[FLMV]-x-[IMV]
	P	0.29	[GSTEQKRV]-Q-[LIVT]-[VAF]-[SAGQ]-G-{DG}-[LIVMNK]-{TK}-x-[LIVMFY]-x-[LIVMFYA]-[DENQKRHSIV]
Gelsolin	A	1	D-[DS]-[IV]-M-[ILMV]-L-D-[AST]-[GW]-[DN]
Huntingtin	A	0.94	L-Y-[GK]-E-I-K-[KR]-N-[AG]-[AN]
Insulin	A	0.95	L-C-G-x-[DGHMNPS]-L-x(0,1)-V-x(5,6)-C-x(3)-G
	P	0.49	C-C-{P}-{P}-x-C-[STDNEKPI]-x(3)-[LIVMFS]-x(3)-C
Islet Amyloid Polypeptide	A	1	S-[HNRS]-N-x(1,2)-G-[APT]-[AIV]-[FL]-x-[PS]-[PT]-[DKNS]
	P	0.33	C-[SAGDN]-[STN]-x(0,1)-[SA]-T-C-[VMA]-x(3)-[LYF]-x(3)-[LYF]
Lactadherin	P	0.15	[GASP]-W-x(7,15)-[FYW]-[LIV]-x-[LIVFA]-[GSTDEN]-x(6)-[LIVF]-x(2)-[IV]-x-[LIVT]-[QKMT]-G
Lactoferrin	A	1	P-V-[AT]-E-A-[EKQR]-[NS]-C-[HY]-L-A-x-A-P-[NS]-H-A-V-V-S
	P	0.38	[DENQ]-[YF]-x-[LY]-L-C-x-[DN]-x(5,8)-[LIV]-x(4,5)-C-x(2)-A-x(4)-[HQR]-x-[LIVMFYW]-[LIVM]
Lysozyme	A	1	G-[ILV]-[FL]-[EQ]-[IL]-N-[DNS]-x(2)-W
	P	0.95	C-x(3)-C-x(2)-[LMF]-x(3)-[DEN]-[LI]-x(5)-C
Prolactin	A	1	R-D-S-x-K-[IV]-[DK]-[NST]-[FY]-L
	P	0.44	C-x-[STN]-x(2)-[LIVMFYS]-x-[LIVMSTA]-P-x(5)-[TALIV]-x(7)-[LIVMFY]-x(6)-[LIVMFY]-x(2)-[STACV]-W
Serum amyloid A	A*	0.96	N-x(1,4)-D-x(3)-[HRY]-[AG]-[PR]-G-[GNS]-x-[DEW]-A-[AQ]-[EKQR]
	P^+^	0.95	A-R-G-N-Y-[ED]-A-x-[QKR]-R-G-x-G-G-x-W-A
Tau	A*	1	G-S-x(0,1)-D-N-[IMV]-[KNRT]-H-x-P-G-G-G-[EKNS]-[KV]-[KQ]-I-x-[DHTY]
	P	0.40	G-S-x(2)-N-x(2)-H-x-[PA]-[AG]-G(2)
Transthyretin	A	1	C-P-L-[MT]-V-K-[IV]-L-D
	P^+^	0.56	[KH]-[IV]-L-[DN]-x(3)-G-x-P-[AG]-x(2)-[LIVM]-x-[IV]
Prion Protein (PrP)	A	0.99	A-x(0,1)-A-x(0,1)-G-x(0,1)-A-[AIV]-[AGV]-[GKY]-x-[AILMV]-x-[DGR]-x(2)-[LMR]-[GPS]-[HRS]
	P^+^	0.93	E-x-[ED]-x-K-[LIVM](2)-x-[KR]-[LIVM](2)-x-[QE]-M-C-x(2)-Q-Y
Sup35	A	0.97	G-x(0,1)-A-x(1,2)-A-[ADEGKNPQRST]-x-[ADEGKNPQRST]-x-L-V-I-S-[ADEGKNPQRST]-[ADEGKNPQRST]-[ADEGKNPQRST]-G-E-[CFHILMVWY]-E
	P	0.13	D-[KRSTGANQFYW]-x(3)-E-[KRAQ]-x-[RKQD]-[GC]-[IVMK]-[ST]-[IV]-x(2)-[GSTACKRNQ]
Ure2	A	0.97	E-F-P-E-V-Y-K-W-T-K

A, best AMYPdb patterns; P, PROSITE super family signatures; P+, PROSITE family signatures; CF, correlation factor; *, indication that the AMYPdb pattern overlaps the PROSITE pattern.

There are many advantages in describing a protein family using several patterns rather than only one or two, as is done in PROSITE. First, the occurrence of more than one pattern increases confidence that a protein belongs to a specific family. Pattern distribution along sequences can also be used to assess conserved and variable regions in proteins. Indeed, highly specific patterns only describe conserved regions in proteins. Examples of this are the Tau and prolactin protein families. Human Tau protein is characterized by 13 patterns with CF ≥ 0.9, all found in the C-terminal region of the protein and covering barely 14% of the sequence. This suggests that the C-terminal part of Tau is the protein's main domain (indeed it is the microtubule-interacting region). On the other hand, 54 patterns with CF ≥ 0.9 are characteristic of human prolactin, and they are distributed all along the sequence, covering 32% of it. This suggests the presence of numerous important regions, which are likely correlated to the many known biological effects of prolactin.

### Amino acid sequence pattern exploration

#### Signatures of biological interest

Although patterns in AMYPdb were created from precursor proteins, users can easily access signatures matching aggregation features and other biological annotations. Indeed, for each pattern, the AMYPdb interface displays its position in sequences, along with the corresponding UniProtKB features. There are 836 highly specific patterns (CF ≥ 0.9) covering annotated regions in proteins. Among these, 251 patterns match variants associated with keywords such as aggregation, amyloidosis, Alzheimer, Parkinson, and so on (FT variant lines in UniProtKB). We have successfully used AMYPdb for knowledge-rich data mining concerning three amyloid families: transthyretin; tau; and prion.

• In AMYPdb, 97 patterns (CF ≥ 0.9), distributed over the entire sequence of human transthyretin (hTTR), map 31 of the 37 single-site amyloidogenic variants described in UniProtKB. In pattern G-E-[ILV]-H-[EGN]-L-x(0,1)-T-x(3,4)-F-x(2)-G-[ILV]-[IY]-[KR]-[ILV]-E, the 9 underlined amino acids correspond to pathogenic missense mutations in human TTR (positions 73–92). In particular, the variant I88L is associated with an amyloid cardiomyopathy. Interestingly, the multiple sequence alignment available in AMYPdb reveals that leucine exists in the wild-type sequence of seven organisms, including bovine and sheep. Comparative study of these proteins with hTTR could help to understand the effect of the mutation isoleucine/leucine in human disease.

• The human Tau protein sequence deduced from the gene is composed of 757 amino acids. It exists however in the human brain as 6 alternatively-spliced isoforms of 352 to 441 amino acids (Tau-A to F), with each isoform containing 3 or 4 repeat domains (R repeats). Using the AMYPdb search interface, we researched each isoform, and found 8 patterns (*CF *≥ 0.9) matching 1 to 6 isoforms. One of the patterns, P-G-G-G-[KNS]-V-Q-I-[FIV]-[DHNY] is observed in all of the isoforms, and matches "hot spot" regions for nucleation, β-sheet aggregation and fibril formation both *in vitro *and *in silico *[19,33,34]. The pattern is located at the junction between R repeats. It matches 3 regions in human tau (PGGGKVQIVY, PGGGKVQIIN and PGGGSVQIVY), that correspond to 2 kinds of junctions: R1–R3 in Tau-A, Tau-B and Tau-C (3 R repeats); and R1–R2 and R2–R3 in Tau-D, Tau-E and Tau-F (4 R repeats). Moreover, the pattern includes variants described as being involved in tau pathogenicity: N596K, delV597, P618L, P618S and S622N (numbering according to UniProtKB P10306).

• In a recent study, Hamodrakas et al. [21] predicted amyloidogenic determinants in several proteins by combining three methods. In the case of human prion (P04156), the authors pointed out the segments 175–183 (FVHDCVNIT), 209–215 (VVEQMCI) and 242–251 (LLISFLIFLI). Using AMYPdb we searched for amino acid sequence patterns and UniProtKB features matching these segments. Some results are summarized in Table 3. The high density of mutation/modification sites overlapping the first two amyloidogenic segments is intriguing. Indeed, these segments contain both the cysteines involved in the unique disulfide bond between helix 2 and 3, and a glycozylation site involved in prion strain propagation [35]. Although other regions have been shown to be important for prion propagation, susceptibility, and other activities, our observations reinforce the idea that *in silico *investigations are more efficient when they combine several methods, such as sequence pattern discovery, aggregation prediction, bibliographical knowledge, and so on.

**Table 3:** PrP regions including amyloidogenic determinants according to [21]

Segments of PrP sequence	Mutations	Modification sites
SNQNNFVHDCVNITIKQHTF	D178N, V180I, T183A	N-Glycozylation 181–184 Kinase C 183–185 Disulfide 179–214
KMMERVVEQMCITQYER	R208H, V210I, E211Q, Q212P, Q217R	Kinase II 216–219 Disulfide 179–214
SPPVILLISFLIFLIVG	P238S	

Column 1: Human prion protein (P04156), segments 170–188, 204–220, and 237–253. Underlined amino acids are those of column 2. Columns 2 and 3: Pathologenic point mutations and potential protein modifications according to UniProtKB.

#### Sequence patterns conserved in several families

By using fitness scores values in the search interface of AMYPdb, it is possible to discover unexpected relations between families of interest. An intriguing observation of cross-conserved patterns between the huntingtin and prolactin families illustrates this. Although these families do not have any known resemblance at their structural/functional level, there are 4 amino acid sequence patterns in AMYPdb matching these 2 families with Sen > 0.5 and Spe > 0.99. These patterns are described below. No non-amyloid sequence among the more than 2 million stored in UniProtKB (release 6.1) contains these 4 signatures simultaneously.

(1) L-[DEGHKNPQRSTY]-C-x(0,2)-R-[ACDNPST]-[AGSTW]-x-[FIKLMRY]

(2) F-x(2)-[LV]-[ILM]-x-[CQS]-x(2)-R

(3) L-x(0,1)-T-x(2,3)-D-[KS]-[DEHY]

(4) L-[DEGHKNPQRSTY]-x-L-[DEHKNQR]-C-[DEGHKNPQRSTY]-x(2)-[DEHKNPQY]

The patterns are distributed all along the sequences. In human huntingtin (3,144 residues) the patterns N°1 to N°4 match respectively at position 212, 1501, 2092, 2789. Positions in human prolactin (227 residues) are 91, 108, 200, and 214 for patterns N° 3, 2, 1 and 4 respectively. Two of those patterns match known structural/functional features in human huntingtin and human prolactin. Pattern N°1 is located in the first "HEAT repeat" of huntingtin, belonging to the N-terminal part of the fragment found in amyloid aggregates [36]. The patterns N°1 and N°4 contain cysteines known to be involved in disulfide bridges in prolactin. They are located in the 4th α-helix of prolactin, already established to be part of the site of interaction with one of the prolactin receptors [37]. The segment of human huntingtin and prolactin corresponding to pattern N°2 is located in the middle of each protein and is predicted by the TANGO algorithm to be a β-aggregating segment [19]. To our knowledge, this is the first time that patterns have been described as shared between huntingtin and prolactin.

Although it seems unlikely that these results were due to chance, we searched for another pattern to confirm our observations. We used PRATT with a new set of 99 sequences, corresponding to all full-length huntingtin and prolactin proteins. We discovered a new highly specific pattern (N°5), R-[DV]-S-x-K-x(2)-[ANSTV]-x(3)-[FILV]-[AGL]-x-[ACS], conserved in 100% of the data set (Sen = 1). In a recent version of UniProtKB (release 10.5, containing more than 4.7 million sequences), this pattern retrieves new prolactin and huntingtin sequences, and only about 50 false positive sequences ones. Pattern N°5 is located at position 710 and 205 in human huntingtin and prolactin respectively, and includes a potential serine phosphorylation site.

This process can be applied to other families. However, it is clear that the quality of a prediction depends on the quality and number of patterns found in common. Experimental work should be undertaken to confirm our observations and to further understand the functional/structural significance of the conserved motifs shared between the huntingtin and prolactin families. These could reveal interaction sites with common cofactors such as chaperones, or common motifs involved in aggregation processes.

#### Pattern repeats

Amino acid repeats play an important structural role in proteins and are often associated with diseases. This is the case with huntingtin, which shows a polyglutamine tract in its N-terminal part. However, repeats are not limited to single amino acids, but can include domains repetitions [38]. For example, repeats are thought to be involved in PrP prionization in mammals, since birds, reptiles, fish and amphibians do not show the same domain architecture [39,40]. In AMYPdb, 41 distinct patterns cover the N-terminal domain of PrPs. We observed that the amino acid sequences of various patterns and their number of occurrences is closely linked to the phylogenetic differences described above, such as for the pattern: G-[GHKQRY]-[GNPSTY]-x-G-[GHQY]-G-x(0,3)-G-[QSWY]-[GHNPQ]-[GHPQRS]-[GNPQSTY]-x-[AGHNST]. In species from fishes to birds (which PrP is not demonstrated to be pathogenic), only 1 occurrence of this pattern was found in the N-terminal region while it is repeated 2 to 5 times in mammalian PrPs. The repetition might therefore act to facilitate the structural conversion of PrPc into PrPsc.

## Conclusion

In this paper, we present a knowledge database dedicated to amyloid precursor proteins and their amino acid sequence signatures [1]. Our work sheds light on the signatures that best describe each amyloid family. Moreover, we have extracted several patterns of interest to demonstrate how users can easily take advantage of the database for their own research. Note that because there are only sparse data on sequences which can form fibrils in vivo, especially non-human organisms, we cannot yet automatically predict aggregation regions. In the future we will continue to enrich the database with new families and new functionalities, ensuring that AMYPdb will remain a reference tool for researchers interested in bioinformatic approaches to protein misfolding and aggregation. A wiki system available in the "identity card" of the proteins allows experts to add high quality data.

## Methods

### Implementation and structure of the database

**Figure 1:**
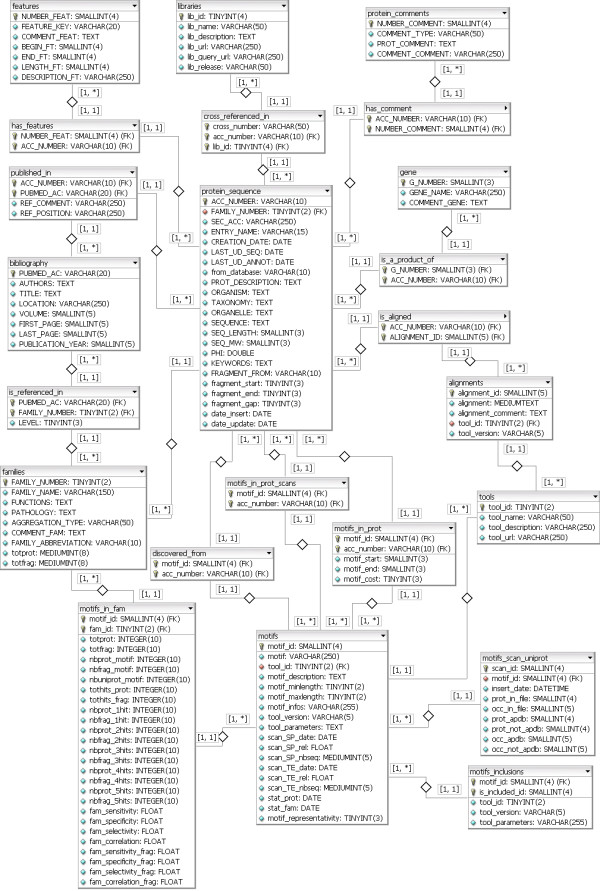
Database structure diagram.

AMYPdb is a MySQL relational database. The web-based interface was created using PHP and JavaScript, and relies on a modified version of the e107 content management system. The data are stored in 23 tables and occupies nearly 4 GB of disk space (Figure 1). The central table contains general information relevant to protein sequences. This is linked to additional tables containing two kinds of data: information collected from public libraries; and the results of protein sequence analysis. This data organization is suitable for complex queries, such as the extraction of amino-acid signatures matching the regions involved in aggregation.

### Raw data

**Figure 2:**
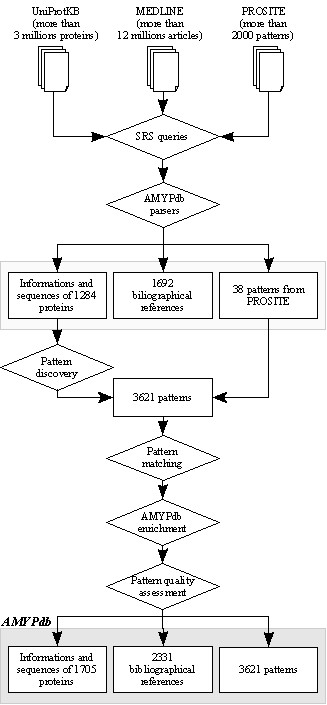
AMYPdb data flow.

The data flow is described in Figure 2. Raw data were extracted from three main public databases: protein files from UniProtKB (release 3.2); references from MEDLINE; and known patterns from PROSITE. We used the Sequence Retrieval System (SRS) program implemented at the European Bioinformatics Institute [41]. SRS has the advantage of possessing a unique interface for interrogating multiple databases. In addition, results can be saved in eXtended Markup Language (XML) format, thus facilitating the exchange and manipulation of large amounts of data between different programs. UniProtKB and MEDLINE were searched using keywords describing the amyloid protein families. The keywords used were mainly protein and gene names commonly used in the amyloid research field. Several phylogenetically distant sequences of each family were then submitted to ScanProsite [42]. This program scans protein sequences for the occurrence of signatures stored in the PROSITE database. The accession numbers of PROSITE patterns were then queried in SRS. All retrieved XML files were imported into AMYPdb. After this first selection step, we obtained a catalog of 31 amyloid families (Table 1), containing 1,284 amyloid proteins, 1,692 references and 38 PROSITE patterns. Data stored in AMYPdb are those of precursor proteins, amyloidogenic peptides and partial sequences. In this first version of AMYPdb, no immunoglobulin light/heavy chains have been stored, due to the high sequence diversity of those proteins.

### Amino acid sequence pattern discovery

#### Pattern discovery

We employed the commonly-used software PRATT, developed to extract conserved patterns in a set of unaligned protein sequences [43]. The "advanced PRATT" version, accessible at OUEST-Genopole^® ^[44], allows users to specify amino acids clusters, thus orienting the discovery of interesting patterns. We selected most of the default parameters of the program, and limited the maximum pattern length to 20 amino acids. This choice is in agreement with data from the recent literature, which show that short protein stretches may be involved in the self-assembly process of amyloid proteins [45].

Various analyses were carried out with "advanced PRATT" using the default clusters, based on the physico-chemical properties of amino acids. Moreover, we defined two other sets of clusters, corresponding to criteria related to amyloid aggregation. The first set was designed based on the ability of amino acids to either form beta sheets (CIFTWYV), alpha helices (AREQLK) or other secondary structures (NDGHMPS) [46]. The second set was based on whether amino acids are found at protein-protein interfaces (CHILMFWYV) or not (ARNDEQGKPST) [47,48]. A refinement parameter was systematically tested (on and off). When the parameter was switched on, ambiguous pattern positions were generalized using the groups of similar amino acids. Among the 31 known amyloid families, 9 could not be submitted to pattern matching because of their small number of known sequences. On the other hand, a few families had enough known sequences to design several data sets. Finally, we applied the pattern discovery method to 42 sets of sequences using 6 parameters, and selected patterns matching 100% of the sequences of each set. Thus, as described in Figure 2, and including the 38 PROSITE patterns, AMYPdb contains 3,621 patterns related to amyloid protein families.

#### Pattern matching

In addition to pattern comparison, we also scanned the UniProtKB database (release 6.1) for sequences matching any of the 3,621 patterns. To do this, we used WAPAM [44], specifically developed to parse a list of amino acid patterns and to search for those patterns in sequence databases. Compared to other pattern-matching tools, WAPAM has several advantages. It has no limit in the pattern's length, flexibility, or indetermination. It also uses Rdisk technology, a specialized architecture that can highly accelerate a search. Using WAPAM with Rdisk, the scan of UniProtKB for the 3,621 patterns took less than 15 hours, instead of the estimated 2,000 hours it would have taken without it. UniProtKB returned 267,490 sequences matching AMYPdb patterns, although the number here is underestimated due to WAPAM's retrieval limitation. The UniProtKB files of these sequences were stored as a non-amyloid group in AMYPdb and were used for the classification procedure described below.

### Database updating procedure

Since the content of UniProtKB was evolving during the various development phases of our project (631,592 entries added from release 3.2 to 6.1), we updated AMYPdb by semi-automatically sorting the 267,490 sequences extracted with WAPAM. From this group, we picked out 421 protein sequences matching highly specific AMYPdb patterns, and we assigned these sequences to the corresponding amyloid families. This updating procedure increased the AMYPdb sequence group to 1,705 members (1,063 full-length sequences and 642 fragments, Figure 2), and leaving 267,069 sequences in the non-amyloid protein group.

### Pattern quality

To measure pattern performance, we used three fitness scores commonly used in classification problems: sensitivity (Sen); specificity (Spe); and correlation (CF) [49,50]. The scores of the 3,621 patterns were calculated for each family, using only full-length sequences. For each pattern, true positives (TP) and false negatives (FN) are sequences of a family respectively either matching or not matching the pattern. False positives (FP) are either amyloid sequences not belonging to the considered family but which match the pattern, or non-amyloid sequences matching the pattern. True negatives (TN) are non-amyloid sequences not matching the pattern. Therefore, when a pattern is specific for one amyloid family, it has high Sen, Spe and CF scores for that family and low scores for other amyloid families. When a pattern is conserved in several amyloid families, the Spe of the pattern remains high for each family, but Sen and CF scores can decrease dramatically. Due to calculation limitations, non-amyloid sequences were obtained from 1 of 3 data sets: the 2,032,835 proteins of UniProtKB was used for patterns matching less than 5000 non-amyloid proteins; the 267,069 non-amyloid sequences resulting from the WAPAM search was used for patterns for matching between 5,000 and 10,000 non-amyloid proteins; and a random pool of 50,000 proteins was used for patterns matching more than 10,000 non-amyloid proteins.

For each pattern, sensitivity corresponds to its ability to describe an amyloid family, while specificity corresponds to its ability to discard proteins not belonging to the amyloid family. The Pearson-Mathews correlation coefficient measures the global prediction accuracy by which a pattern describes a protein family. A CF of +1 indicates that the pattern perfectly differentiate the amyloid family from other amyloid families and non-amyloid proteins.

$$\begin{array}{l} Sen=TP/(TP+FN)\\ Spe=TN/(FP+TN)\\ CF=\frac{\left((TP\times TN)-(FP\times FN)\right)}{\sqrt{\left(TP+FP)\times (FP+TN)\times (TN+FN)\times (FN+TP\right)}} \end{array}$$

### Amyloid classification

The accuracy of hypotheses deduced from bioinformatic methods strongly depends on data sets quality. In the present study, the sequence sets used in the pattern discovery method were those of protein families. In order to facilitate sequence extraction, especially for the discovery of patterns link to misfolding and aggregation, all the proteins were sorted into one of the five following quality categories:

• Amyloid *in vivo*: the precursor protein, or a specific sub-segment, forms fibrils in human, or animals, or is a yeast prion. Proteins of this class are unambiguously described in literature and are identified by specific keywords in UniProtKB ("Amyloid" or "Prion").

• Amyloid *in vitro*: the polypeptide forms fibrils under experimental conditions.

• Amyloid *in silico*: the polypeptide forms fibrils using computational techniques, including protein threading and molecular dynamics simulations.

• Putative amyloid protein: the protein is a member of an amyloid family, but the amyloid properties of that specific member were not assessed.

• Unclassified protein: the protein family does not fulfill the definition of amyloid [4], but sparse data show that at least one member of the family shares some amyloid properties.

At the present time, the classes 2 and 3 are empty. Experts are welcome to contribute to relevance of biological information by changing the status of the proteins using the Wiki system.

## Authors' contributions

SP designed and built the MySQL database and the web interface. Both SP and CD carried out data analysis and drafted the manuscript. ALB participated in the final version of the database, including interface improvements and assignment of the proteins to the amyloid classification. All authors read and approved the final manuscript.
